# Anti-EGFR Therapy: Mechanism and Advances in Clinical Efficacy in Breast Cancer

**DOI:** 10.1155/2009/526963

**Published:** 2009-04-14

**Authors:** John F. Flynn, Christina Wong, Joseph M. Wu

**Affiliations:** Department of Biochemistry and Molecular Biology, New York Medical College, Valhalla, NY 10595, USA

## Abstract

This review will focus on recent advances in the application of antiepidermal growth factor receptor (anti-EGFR) for the treatment of breast cancer. The choice of EGFR, a member of the ErbB tyrosine kinase receptor family, stems from evidence pinpointing its role in various anti-EGFR therapies. Therefore, an increase in our understanding of EGFR mechanism and signaling might reveal novel targets amenable to intervention in the clinic. This knowledge base might also improve existing medical treatment options and identify research gaps in the design of new therapeutic agents. While the approved use of drugs like the dual kinase inhibitor Lapatinib represents significant advances in the clinical management of breast cancer, confirmatory studies must be considered to foster the use of anti-EGFR therapies including safety, pharmacokinetics, and clinical efficacy.

## 1. Introduction

Despite the availability of a new array of biomarkers and a widely adapted clinically relevant/treatment-oriented approach of classifying breast cancer cases over the last decade, categorization of breast cancer is an ongoing challenge which is being revisited more frequently by the scientific community. The goal is to fine-tune the diagnostic assignment of breast cancer cases with the hope that this will adequately address and improve the effectiveness of selecting treatment modalities, particularly in regard to the choice of use of monoclonal antibodies (MoAbs) and small molecule tyrosine kinase inhibitors (smTKIs) against EGFR, a clinical strategy collectively referred to as anti-EGFR therapy. EGFR is a member of the ErbB/HER family of tyrosine kinase receptors, which also includes its well-documented family member ErbB2, clinically referred to as HER-2/neu. Anti-EGFR therapy has found application for cases from all three major breast cancer subclasses, respectively, the hormone-sensitive/insensitive group, the ER+/− and HER-2/neu+/− groups, and the basal-like/triple negative (−) group. Of note, HER-2/neu may also be a genetic biomarker since it has a more significant correlation with a selective HER-2 (+ve) population of breast cancer cases than EGFR. Preliminary studies show that anti-EGFR therapy has moderate clinical efficacy not only on EGFR-expressing cells, but on HER-2-expressing and -overexpressing cells as well, suggesting that the treatment outcome may depend on the expression and responsiveness of the heterodimerization of HER-2 with EGFR. Although both EGFR and HER-2 (+ve) are favored biomarkers of efficacy in many ongoing anti-EGFR clinical studies, their expression is not sufficiently robust as a prognosticator for clinical outcomes and should not be singularly used as a criterion for evaluating the responsiveness of breast cancer cases to anti-EGFR treatment regimens [1]. Tumor targets for anti-EGFR therapy include early and advanced stage, and metastatic breast cancer as well as an array of other solid tumors that are not part of this review; data from recent studies suggest that various anti-EGFR/TKI combinations may not only treat but also lower progression rates of these forms of cancer.

The primary focus of this article is to review and summarize recent advances in anti-EGFR therapies in order to generate a clinically relevant profiling system; a complementary objective is to relate the structure of EGFR with its downstream signaling mechanisms particularly in the context of inhibition by administered anti-EGFR therapies. Database search engines like MEDLINE, PubMed, Scopus, and ENTREZ were used, and the articles were selected according to the criteria: (i) anti-EGFR therapy and clinical efficacy in breast cancer, (ii) publications from 1998–2008, and (iii) using reviews/conferences/special reports/randomized clinical trials/phase II and III trials/general research articles. It is hoped that reviews like this can help to elucidate the mechanisms involved in anti-EGFR therapy as well as define relationships between the overexpression of EGFR and other biomarkers of breast cancer. Recent data regarding responsiveness to combination and multiregiment chemotherapies may also provide insight on the mechanism and activity of anti-EGFR therapies, specifically that of the dual kinase inhibitor, Lapatinib (GW572016), which is capable of targeting both the EGFR and HER-2/neu tyrosine kinases that are often overexpressed in breast cancer cells [4].

## 2. EGFR and Its Role in Breast Cancer

**Figure 1:**
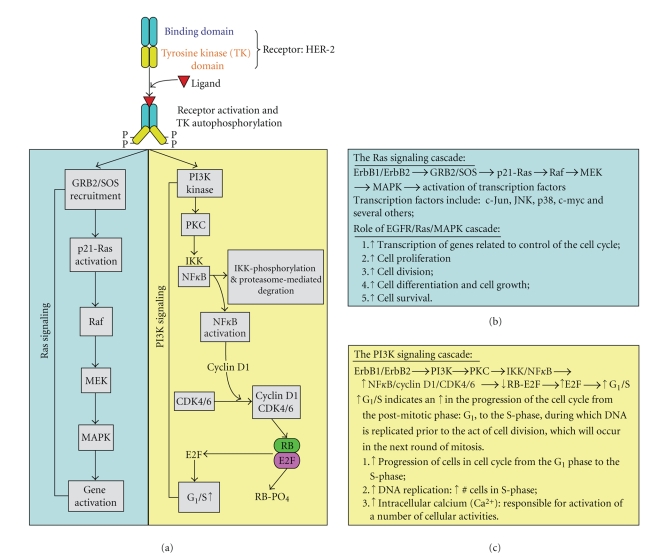
The EGFR Signaling Pathway. (a) Upon EGF-ligand binding to the EGFR there is subsequent dimerization (homo- or hetero-) and tyrosine kinase residue auto-/transphosphorylation of dimer partners, which in turn initiates the actual downstream signaling pathways. (b) Ras signaling cascade in tabulated form. (c) PI3K signaling cascade in tabulated form.

EGFR is a member of the EGFR/ErbB/HER family of Type I transmembrane tyrosine kinase receptors, which includes ErbB1/HER-1 (EGFR itself), ErbB2/HER-2/neu, ErbB3/HER-3, and ErbB4/HER-4. The ErbB receptors play an essential role in organ development and growth by regulating both the differentiation and morphology of cells and tissues. However, specific members, most notably EGFR, are frequently overexpressed, and this aberrant expression and the signaling event it elicits induce erroneous development and unrestricted proliferation in a number of human malignancies including breast cancer [5]. Members of the ErbB gene family, respectively, ErbB1, ErbB3, and ErbB4 can be activated by various growth factor ligands, for example, the epidermal growth factor (EGF). In contrast, no known ligand has been demonstrated for ErbB2/HER-2/neu, despite that it still plays an integral role in several signaling pathways as well as tumorigenesis. Activation of EGFR inevitably involves homo- or heterodimerization of EGFR with another EGFR molecule, or a different member of the ErbB family (e.g., HER-2), which in turn induces the amplified signaling cascade (Figure 1). Increased activation of EGFR and/or HER-2 will eventually result in uncontrolled proliferation, a hallmark of cancer cells. Additionally, the cells harboring overexpressed EGFR or improper regulation of EGFR activation may decrease apoptosis, increase metastasis and even angiogenesis. Dysfunctional EGFR-signaling networks are reportedly present in a cohort of breast carcinomas with poor prognosis [5, 6].

To better understand the role of EGFR in breast carcinogenesis, the aforementioned relationship will be analyzed in several parts. First, it is important to thoroughly investigate thesignaling pathway and mechanism of EGFR to properly examine the correlation that exists between breast cancer and anomalous EGFR expression; in this case, scrutiny of the structure of EGFR and the role it plays in cell signaling is imperative. Secondly, anti-EGFR therapies for breast cancer either in ongoing clinical phase testing or already FDA-approved are reviewed or summarized in the tables, with focus directed to specific developments and progress in clinical efficacy in recent years. Examples discussed in detail in Section 3 include Cetuximab, a monoclonal antibody against EGFR and the most widely used anti-EGFR therapy in solid tumor treatment regimens; Lapatinib, an innovative small molecule tyrosine kinase inhibitor of EGFR with a unique dual-TKI inhibitory activity against both EGFR and HER-2/neu, which shows improved clinical efficacy and has heightened the expectation for new breast cancer therapies. Although many recent studies have demonstrated a beneficial role of Lapatinib used in combination with anti-EGFR therapy, only selected examples will be reviewed to illustrate how Lapatinib may be strategically explored to improve our understanding of the synergy resulting from its use associated with anti-EGFR therapy.

### 2.1. EGFR Structure and Signaling Pathway Mechanism

**Figure 2:**
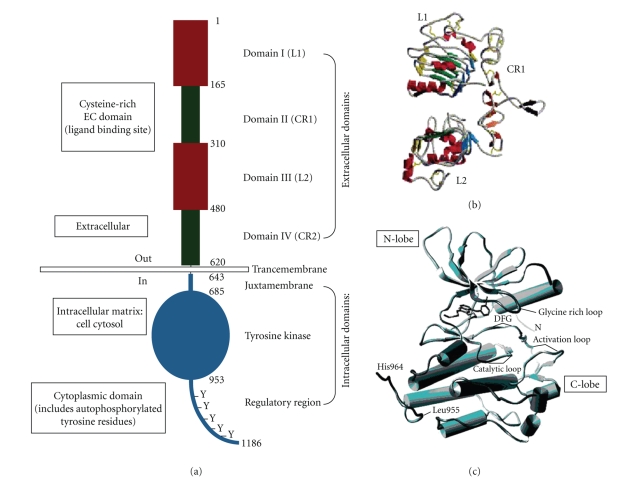
(a) Basic Structure of EGFR demonstrating relevant domains. *(I) The extracellular domains*: (1) domain I: L1; (2) domain II: CR1; domain III: L2; domain IV: CR2. *(II) Transmembrane domains*. *(III) The* intracellular *domains* (1) juxtamembrane domain; (2) tyrosine kinase domain; (3) regulatory region domain. The phosphorylation of several substrates by the tyrosine kinase domain of the EGFR receptor is responsible for activating the various signaling cascades seen in Figure 1. (b) *Structure of domains I–IV of EGFR* (no ligand bound). Note the “protruding loop” in domain II (CR1) directed away from the *C-shaped region* of the ligand-binding zone formed by domains I, II, and III. (c) The tyrosine kinase domain of EGFR showing the N-lobe and C-lobe flanking the activation loop and active site cleft [2, 3].

The EGFR and the activated signaling cascades it elicits play an integral role in the mechanism and efficacy of anti-EGFR therapies (Figure 1). Examination of the EGFR structure (Figure 2) provides a contextual framework for the inception and development of two major strategies of anti-EGFR therapy, respectively, anti-EGFR monoclonal antibodies and small molecule tyrosine kinase inhibitors (smTKIs). Examples of monoclonal antibodies for EGFR are found in Cetuximab and Trastuzumab. Anti-EGFR drugs belonging to smTKIs and in clinical trials include Erlotinib, Lapatinib, and Gefitinib.

#### 2.1.1. Molecular Analysis of EGFR Structure

**(1) Ectodomain: Ligand-Binding Domain**

Domains I–IV make up the EGFR ectodomain, a 621-kDa structure responsible for ligand binding and dimerization, both of which are considered molecular antecedents for the induced conformational changes required for the activation of the internal tyrosine kinase. Although the EGFR ectodomain was first crystallized in 1998, the detailed structure of the EGFR ectodomain dimer bound to ligand EGF was not resolved until 2002 and provided information on a completely novel and unexpected mode of activation for the EGFR signaling pathway involving the dimerization process [2, 7–9].

Domain I or L1 (where L: leucine-rich domain) shares sequence and structural homology with the Domain III or L2, both of which are involved in ligand binding based on site directed mutagenesis and deletion mutation studies [10]. Domains II and IV, also referred to CR1 and CR2 reflecting their high content of cysteine residues and the potential for forming intradomain disulfide bonds, are important in facilitating the overall conformational change induced by binding of the ligand to EGFR. The CR1 also contains a “protruding loop” capable of bending in a relatively straight, ligand-binding site of EGFR (Figure 2(b)). This flexible molecular feature presumably enables binding of the ligand between the two L domains and at the same time permits contacts to be made with the “protruding loops” in CR1.

**(2) Transmembrane and Juxtamembrane Domains**

The transmembrane domain consists of 23 amino acids and plays an important role in anchoring the receptor to the lipid bilayer of the cell. More than 50% of the transmembrane domain is localized in caveolae or lipid rafts [10], through posttranslational modifications, such as N-linked glycosylation, resulting in enrichment of EGFR in defined locality of the membrane and hence faster receptor dimerization following binding of the ligand [11]. Adjacent to the transmembrane domain facing intracellularly is the juxtamembrane domain which is believed to regulate various functional aspects of EGFR including control of the tyrosine kinase activity, downregulation of the EGFR, ligand internalization, and receptor sorting. Of note, this domain also has binding motifs that allow it to interact with second messengers like calmodulin [10].

**(3) Tyrosine Kinase Domain**

The tyrosine kinase domain (TKD) is essential for the functional activation of the receptor and consequently the induction of the EGFR signaling pathways for the control of cell division and proliferation. The TKD has a bilobate arrangement marked by an N-lobe, an activation loop, and a C-lobe [3]. This molecular configuration accommodates binding of the substrate and ATP at the active site, enabling substrate phosphorylation to occur in concomitance with the hydrolysis of ATP (Figure 2(c)). The TKD contains important tyrosine (Y) residues that can assume various states of phosphorylation/dephosphorylation. Knockdown or deletion studies of the ectodomain suggest that it regulates the dimerization as well as prevent constitutive activation of the tyrosine kinase. Binding of the ligand to the ectodomain relieves some of the steric hindrances normally imposed on the tyrosine kinase activity, resulting in activation. Site-directed mutagenesis or deletion analysis in the TKD shows that it is involved in EGFR dimerization, auto- and transphosphorylation and also activation of the signaling cascades, all of which have been exploited in the development of small molecule tyrosine kinase inhibitors (smTKIs) for targeting EGFR in various cancer types including breast cancer.

**(4) The Activation Loop, Active Site, and C-Terminal Tail Regions of EGFR**

The activation loop of the tyrosine kinase of EGFR is quite distinct from other receptors harboring tyrosine kinases. Namely, whereas most receptor TKDs require phosphorylation for tyrosine kinase activation, this does not appear to be the case in EGFR. For example, the phosphorylation of Tyr^845^ has little affect on the EGFR kinase activity [3], possibly due to a conformational arrangement that directs the activation loop away from the active site rendering it refractory to the state of phosphorylation of the receptor tyrosine kinase. Activation of the EGFR tyrosine kinase phosphorylates numerous targets, including itself (autophosphorylation), a different EGFR (homodimerization), HER-2/neu of the ErbB gene family (heterodimerization), and nonreceptor substrates such as Grb2/SOS, STATs, PLC, and/or PI3K, which in turn initiate the signaling cascades of MAPK/ERK, STAT, PIP_2_, and AKT, respectively. Not surprisingly, therefore, mutations in this region can cause a substantial decrease in kinase activity, an outcome considered desirable in cancer therapy and may underlie the therapeutic efficacy of smTKIs. By binding to the TKD of EGFR, smTKIs may act by sterically interfering with the binding of both the substrate and ATP necessary for phosphorylation, resulting in an overall decreased signaling activity of the EGFR.

Lastly, it is important to mention that the tyrosine kinase activity of EGFR is tightly regulated via its own internal regulatory region located at the C-terminal tail of the structure, which involves the tyrosine residue cluster with the potential of being transphosphorylated during EGFR-dimerization. It is noteworthy that EGFR dimerization induces phosphorylation of several tyrosine residues including Tyr^1069^ Tyr^1092^ Tyr^1110^ Tyr^1116^ Tyr^1172^ Tyr^1197^, creating docking sites for the recruitment of other adaptor molecules and signaling proteins. These attributes suggest that the tyrosine-rich C-terminal tail is a phosphorylable, mobile structure connected to a relatively stationary TKD.

In summary, the EGFR may be divided into two functional substructures. The first one consists of the extracellular ectodomain responsible for ligand binding, dimerization, and the initiation of signal transduction. The ectodomain has been the thematic target of anti-EGFR therapy, vis-à-vis, development of monoclonal antibody directed at the ligand binding region, which inactivates EGFR through competitive inhibition of ligand binding, as well as by inducing overall downregulation of EGFR through increased receptor internalization. Examples include the monoclonal antibodies like Cetuximab and Trastuzumab, which play an extremely critical role in anti-EGFR therapy. Currently, both these two drugs and Panitumumab are the only anti-EGFR monoclonal antibodies approved by the FDA for use in the clinic. The second major functional substructure of EGFR is the tyrosine kinase domain located on the intracellular side of the plasma membrane. This domain plays a key role in the activation of signaling cascades involved in cell proliferation, division, and differentiation; therefore, inhibition of the tyrosine kinase enzymatic activity of EGFR using small molecule TKIs is a clinically relevant treatment option for breast cancer patients.

### 2.2. Breast Cancer and the Signaling Mechanism of EGFR

As a member of the ErbB receptor family, the EGFR plays important roles in cell signaling, proliferation, differentiation, and apoptosis. Signaling is initiated by binding of ligands to the extracellular domain of the EGFR. Six well-characterized ligands of EGFR have been identified, respectively, EGF, transforming growth factor-*α* (TGF*α*), amphiregulin, heparin binding EGF-like growth factor, betacellulin, and epiregulin. Ligand binding induces conformational change resulting in heterodimerization and the activation of the major signaling pathways seen in Figure 1.

#### 2.2.1. Statistics and Etiology of Breast Carcinogenesis

Breast cancer is the most common cancer and a major cause of morbidity and premature loss of life in women worldwide, accounting for approximately 7% of all cancer-related deaths [12]. The highest rates of breast cancer in the world are seen in the United States, where approximately 1 out of every 8 women will develop invasive breast cancer, which is responsible for almost 3% of all deaths in American women [13]. Given the grim statistics, the need for more sensitive and reliable detection methods is obvious. Equally urgent are treatment modalities that are modest in cost, easily compliant, effective, have low to no toxicities, and capable of targeting the multifaceted and heterogeneous nature of breast carcinogenesis.

Currently, there is still lack of understanding of the natural history of breast cancer. It had been hypothesized that lobular carcinoma in situ (LCIS) represented a precursor lesion of invasive cancer, and, based on this, mastectomy was initially recommended [14]. Later studies have shown that the risk of subsequent breast cancer is bilateral. Moreover, it became evident that LCIS is not a premalignant lesion, but rather a marker that identifies women at an increased risk for subsequent development of invasive breast cancer, with the risk remaining elevated even beyond two decades. In a large prospective study from the National Surgical Adjuvant Breast and Bowel Project involving a 5-year follow-up of 182 women with LCIS managed with excisional biopsy alone, eight women developed ipsilateral breast tumors (four with invasive tumors), and three women developed contralateral breast tumors (two with invasive tumors) [15]. Therefore, it remains unclear whether or not LCIS progresses to ductal carcinoma in situ (DCIS) during breast carcinogenesis. On the other hand, DCIS is a bona fide precursor for invasive ductal carcinoma and lacks estrogen receptor (ER) expression. Furthermore, DCIS frequently overexpresses mutated p53, HER-2/neu, and EGFR, all of which show some clinical correlation with resistance to hormone therapy and increased risk for the development of invasive, metastatic breast cancer. Patients with ER(+ve)/PR(+ve) disease usually respond more favorably to hormonal therapy (as compared to individuals with ER(−ve)/PR(−ve) status), presumably in part due to the overexpression of HER-2 and EGFR in ER(−ve) cells that provide “acquired growth stimulation autonomy.” These findings suggest that strategies cotargeting HER-2 and EGFR expression or their functions might have therapeutic and preventive potentials particularly in ER(−ve) breast carcinoma cases.

#### 2.2.2. Expression/Function of HER-2/EGFR and Signaling in Breast Carcinogenesis

The EGFR gene is frequently altered by gene amplification or overexpression at the mRNA and protein levels in sporadic breast cancer cases. Numerous polypeptide ligands sharing an EGF-like motif have been identified and shown to be capable of inducing EGFR dimerization with different kinetics, eliciting signals of variable durations, and coupled signal transduction to specific sets of cytoplasmic proteins. In principle, therefore, this “ligand-initiated receptor-mediated signaling-executed” molecular relay system might generate a large combinatorial set of biological readouts with enormous potential for diversification, fine tuning, and stringent control of cellular functions and responses. Of note, the HER-2/EGFR has been proposed to act as a master regulator of a signaling network that drives breast carcinoma epithelial cell proliferation; HER-2 gene amplification was observed in 92% of breast cancer specimens and overexpression of HER-2 at the mRNA, and protein levels have been correlated with cancer virulence, resistance to therapy, and poor prognosis. As discussed, each member of the EGFR gene family has a multifunction structural organization comprised of an extracellular ligand-binding/interacting domain connected by a transmembrane span to an intracellular kinase domain. In an uninduced state, the EGFR is organized such that the autoinhibitory loops flanking the kinase active site sterically inhibit it from binding substrates. Binding to ligands induces EGFR homo-or heterodimerization concomitant with its autoactivation by a transphosphorylation mechanism involving specific tyrosine residues located in the intracellular domain. In turn, phosphorylated EGFR undergoes conformational changes that create additional docking sites for adaptor proteins, kinases, and intracellular messengers. Therefore, a tightly regulated, dynamic equilibrium presumably exists between inhibited, activated, monomeric, and dimeric EGFR in order for proper cell signaling to ensue. If any one of these mechanisms goes awry, the results can be fatal to the cell and often can be fatal to the organism. The same considerations may well contribute to the observed clinical efficacy or lack thereof in EGFR-targeted therapies.

The realization that HER-2 is a master regulator of a signaling network that drives epithelial cell proliferation identifies this protein as a target for cancer therapy. When overexpressed, the HER-2 protein may be constitutively active, that is, signaling from the receptor occurs by a ligand-independent manner. Under these conditions, growth-promoting signals may be continuously transmitted into the cells in the absence of ligand. As a result, multiple intracellular signal transduction pathways become activated, resulting in unregulated cell growth and, in some instances, oncogenic transformation. Figure 1 depicts some of the signaling pathways elicited in response to ligand binding to EGFR and either EGFR/EGFR homodimerization or EGFR/HER-2 heterodimerization reactions. It also demonstrates the cascade of events resulting in the transmission of signals into the nucleus and subsequent cell proliferation and gene activation. The intracellular signaling pathways of EGFR and HER-2 are thought to involve Ras-MAPK, and PI3K-, PKC-, NF*κ*B-mediated pathways. Many clinical trials have observed a poor clinical outcome and shortened survival time for women whose breast tumors have HER-2 amplification. An inverse correlation of ER and HER-2 levels between ER(+ve) and ER(−ve) breast cancer cells has been demonstrated, which probably accounts for the development of tamoxifen resistance in breast cancer cells.

#### 2.2.3. Signaling Cross-Talk and Acquisition of Endocrine Resistance

Multiple lines of evidence implicate breast cancer development and progression as under the control of steroid hormones, in particular estrogens, via their interaction with estrogen receptors (ERs) and cross-talk of ER with receptors including EGFR [16–18]. The classical mechanism of ER signaling involves binding of estrogens to intracellular ER, triggering a multitude of events that culminate in altered transcription of estrogen-responsive genes. In sequence, protein synthesis occurs resulting in cell proliferation, angiogenesis, breast cancer growth, progression, and metastasis [19–21]. The ER-induced signaling mechanism coupled with the fact that well over two thirds of breast cancers exhibit high expression of ER, have provided the rationale for preventing and treating breast cancer by estrogen antagonism, highlighted by the discovery of tamoxifen. By selectively modulating the ER, tamoxifen is considered the mainstay of estrogen antagonist therapy and among the most effective systemic treatmentfor women with ER-positive breast cancer at all stages today [21]. A serious obstacle, however, is intrinsicor acquired resistance to endocrine agents. Manypatients present with primary (de novo) resistance to endocrinetherapy, despite high tumor levels of ER, and all patients withadvanced disease eventually acquire resistance [22].

What underlies the refractoriness to endocrine therapies? A number of possible explanations may be considered. For example, in addition to the aforementioned activation of intracellular ER for transcription, estrogens have also been shown to bind membrane-associated ER [20]. Evidence also exists on ER activation by a ligand-independent but growth factor-dependent kinase-mediated mechanism [16]. Important contributing factors for resistance to endocrine therapy include the levels of both ER and ER coregulatory proteins, amplified extra- and intracellular signaling from growth factor-mediatedpathways, as well as cross-talk between the ER pathwayand other growth factor and kinase networks [16–18]. Other mechanisms may involve amplification and/or mutations of key proteins involved in cross-talk, as well induction of promiscuity and/or antagonism to therapeutic agents through mutational and posttranslational modification events [21]. It is possible that aberrations and dysfunctions in these and other mechanisms may occur with increasing frequency during the development of the endocrine-resistant phenotype. Delineation of the interplay between the estrogens, ER, and ER cross-talk with receptors like EGFR will be an important diagnostic and prognostic objective in anti-EGFR therapy. Similarly, discovery and development of novel agents that can reverse resistance by targeting the ER and its downstream signaling events, or by selective modulation of the ER : EGFR cross-talk might improve therapeutic response rates.

In summary, identifying the factors and pathways responsible for endocrine resistance and defining ways to overcome it are research gaps in need of further study and will remain important diagnostic and therapeutic challenges in the continuing war to better manage and treat breast cancer.

#### 2.2.4. Breast Cancer Treatment Using Multitarget Strategy Related to HER-2 Signaling

The amplification of the HER-2 gene and overexpression of the HER-2 protein is frequently observed (10–40%) in human breast cancer patients [23] and has been suggested to associate with tumor aggressiveness, prognosis, and responsiveness to hormonal and cytotoxic agents. These observations suggest that HER-2 is an appropriate target for tumor-specific therapies, some of which are listed as follows.

1. A humanized monoclonal antibody against HER-2, rhuMAbHER-2 (Trastuzumab), is already approved for clinical use in the treatment of patients with metastatic breast cancer. Some forms of HER-2 overexpressing breast tumors can be successfully treated using antireceptor monoclonal antibodies, *for example*, Herceptin. However, because multiple proteins are involved in growth-signaling pathways, development of a uniformly active therapy may be strategically challenging. Herceptin inhibited the P13K-dependent pathway, and not the MAPK pathway. Also, blockade of HER-2 function alone without the interception of the committed, associated downstream events may restrict the effectiveness of therapeutic interventions.
2. Tyrosine kinase inhibitors, such as emodin, which block HER-2 phosphorylation and its intracellular signaling.
3. Heat shock protein Hsp90-associated signal inhibitors, which induce degradation of tyrosine kinase receptors, such as HER-2.

## 3. Classes of Anti-EGFR Therapy

### 3.1. Advances in the Clinical Efficacy of Anti-EGFR Therapies for Breast Cancer Treatment

The race for a successful breast cancer treatment intensified during the late 1990s and 2000s, resulting in the development of innovative anti-EGFR therapies in the last few years including both monoclonal antibodies (MoAbs) and small molecule tyrosine kinase inhibitors. To systematically analyze and summarize the clinical outcome of these anti-EGFR therapies, it is useful to identify and define key terms used in clinical trials. The relevant key terms can be found in various tables presented below, as appropriate (*see Table* 1).

### 3.2. Monoclonal Antibodies

#### 3.2.1. Cetuximab

Cetuximab is the most commonly used anti-EGFR therapeutic agent for the treatment of solid tumors. Originally developed for treating colorectal cancer (primary: CRC & metastatic: mCRC) and squamous cell carcinoma of the head and neck and not yet approved as therapy for breast cancer, Cetuximab does provide an excellent model for the development of new MoAbs that may one day be used in breast cancer therapy. As a humanized mouse MoAb similar to others currently in development: for example, EMD72000 (Matuzumab) and hR3 (Nimotuzumab), Cetuximab differs from fully humanized MoAbs like Panitumumab, which have a lower incidence of adverse events (AEs) (e.g., rash, diarrhea) [25]. In addition, although Panitumumab blocks ligand-binding to EGFR and causes receptor internalization like humanized Cetuximab, it does not induce degradation of the receptors [25].

#### 3.2.2. Trastuzumab

Trastuzumab is an anti-HER-2 receptor humanized MoAb that has shown significant clinical benefits for the treatment of HER-2/neu(+ve) metastatic breast cancer as a single agent [26]. Phase II study investigated the clinical efficacy and safety of Trastuzumab monotherapy given as first-line treatment once every 3 weeks in woman with HER-2(+ve) metastatic breast cancer (MBC). In 105 patients receiving five cycles of therapy, the overall response rate was 19% and the clinical benefit rate was 33%. Median time-to-progression was 3.4 months (range, 0.6 to 23.6 months).In general, the monotherapy was well tolerated and no significant AEs were reported. The most common treatment-related AEs were only mild-to-moderate rigors pyrexia, headaches, nausea, and fatigue. Tables 2(a) and 2(b) show the clinical efficacy and common AEs of Trastuzumab monotherapy [27].

Trastuzumab has also been shown to improve survival rates after chemotherapy, specifically in the Herceptin Adjuvant (HERA) study [6]. HERA is an international multicenter-randomized trial comparing 1 or 2 years of Trastuzumab treatment with observation alone after standard neoadjuvant or adjuvant chemotherapy in women with HER-2(+ve) node positive or high-risk node negative breast cancer. In an intention-to-treat analysis of a total of 5102 patients, the unadjusted hazard ratio for the risk of death with Trastuzumab compared with observation alone was 0.66 (95% CI 0.47–0.91; *P* = .0115). Overall, the hazard rates were lower for Trastuzumab treatment group after 1 year compared to the observation group [29]. After 1 year of Trastuzumab treatment, there were 218 disease-free survival events and 59 deaths, whereas 321 disease-free survival events and 90 deaths occurred in the control group. Patients with one or more grade 3 or 4 AEs were 11%, however, there were minimal cardiac AEs in the 1 year Trastuzumab group with no reported deaths related to cardiac failures. Therefore, one-year treatment of Trastuzumab after adjuvant chemotherapy has significant overall survival rates and minimal AEs [29].

Trastuzumab in combination with chemotherapeutic agents, specifically paclitaxel and anthracycline (Doxorubicin), has shown significant increases in response rates and disease-free progression (*See Table* 3) [27–29]. The combination prolonged the median time to disease progression from 4.6 to 7.4 months, increased the overall response rate from 32 to 50%, extended duration of response from 6.1 to 9.1 months, and improved 1-year survival times from 68 to 79% compared with chemotherapy alone. The clinical efficacy of Trastuzumab alone and in combination with other chemotherapy options is shown in Table 3. The probability of survival was shown to increase by 25% for 25.4 months with Trastuzumab-*plus*-chemotherapy compared to just 20.3 months for chemotherapy alone. Therefore, it appears that Trastuzumab may sensitize cancer cells to other forms of chemotherapy. AEs were expectedly seen as in other chemotherapy-treated patients with MBC. Thus, the combination of Trastuzumab with chemotherapy (anthracycline or paclitaxel) was active for the treatment of patients with HER-2(+ve) MBC who had not been previously treated for metastatic disease. As expected, the clinical benefits from the treatment with Trastuzumab only apply to HER-2/neu(+ve) breast cancer patients, future studies should be designed using new targeted patient population.

### 3.3. Tyrosine Kinase Inhibitors

#### 3.3.1. Lapatinib

**Figure 3:**
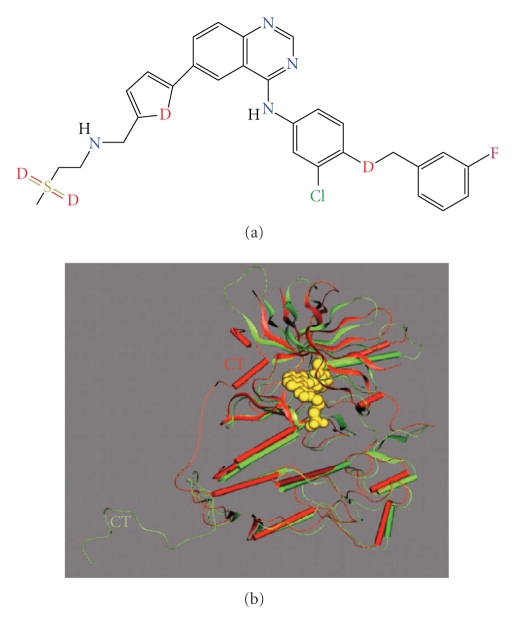
Molecular and crystal structures of EGFR inhibitor Lapatinib and Lapatinib bound and complexed to EGFR ATP-binding pocket, respectively. (a) Molecular structure of Lapatinib (CID208908), an EGFR-ErbB2 inhibitor. (b) Overlay of EGFR in the Lapatinib and Erlotinib complexes. EGFR in the Lapatinib and Erlotinib structures is shown as red and green ribbons, respectively. Lapatinib is shown as a yellow space-filling model. The two proteins were overlaid based on residues in the COOH-terminal domain of the kinase. The COOH-terminal in both structures is CT. Disordered residues in the COOH-terminal tail of EGFR are indicated by a dashed line. The figure was prepared using QUANTA (Accelrys), adapted from Wood et al. [36].

Lapatinib is a member of the orally active small molecules that reversibly inhibit both ErbB1 and ErbB2 tyrosine kinases, which consequently leads to the downregulation of both the MAPK and PI3K signaling cascades responsible for cell proliferation and survival, respectively. As a dual kinase inhibitor, Lapatinib has shown activity in a number of different metastatic and advanced tumor cell lines as well as xenografts and has recently shown positive results in clinical testing as well (*see Figure 3 and Tables* 4(a) *and* 4(b)). Inhibition of ErbB1/EGFR alone using Gefitinib and Erlotinib, examples of anti-ErbB1 smTKIs, has shown mixed clinical efficacy results for MBC [31]. Recent studies have demonstrated that it may be advantageous to inhibit ErbB1 and ErbB2 simultaneously in those patients overexpressing the ErbB2/HER-2/neu gene, which constitutes approximately 25% of all cases of primary breast cancer [32–35]. Interestingly, although both Lapatinib and Erlotinib bind the ATP-binding site of EGFR, only Lapatinib displays the unique dual kinase inhibitory activity. The molecular underpinning for the observed differences awaits further research in the future [36].

Clinical efficacy and safety of Lapatinib as a monotherapy has been recently tested for HER-2-amplified locally advanced cases or MBC [30]. In a total of 138 patients treated with Lapatinib for a median of 17.6 weeks, the overall response rate was 24% and the clinical benefit was 31%. The median time to response was 7.9 weeks, and the progression-free survival rates at 4 to 6 months were 63% and 43%, respectively. Response rates and common AEs are reported in Table 4. The most common AEs were diarrhea, rash, pruritus, and nausea, which were primarily grade 1 or 2 toxicities. This study supports further use of Lapatinib in first-line and early-stage HER-2-overexpressing breast cancer patients [30].

Combination therapy involving Lapatinib has had mixed results as of 2008. For example, despite the fact that Lapatinib combination therapy with Capecitabine has shown success in treatment for HER-2(+ve) advanced breast cancer treatment, a subpopulation of patients often reported occurrences of grade 4 diarrhea, as well as fatigue, headache, and dizziness [40]. The same study eventually reported a discontinuation of treatment (of combined Lapatinib-*plus*-Capecitabine) due to increased occurrence of AEs in 22 women in the combination-therapy group (13%) [40]. However, it was also reported that 18 women in the monotherapy group (12%), also experienced this high frequency of AEs, which appears to be inconsistent with the safety reports for Lapatinib in a number of other sources, which report no reports of any drug-related grade 4 AEs Table 5 [37, 38, 40].

Furthermore, regarding Phase I safety reports for Lapatinib, there were also no reports of drug-related interstitial pneumonitis or cardiac dysfunction that was normally found to be associated with other forms of ErbB-targeted therapies [37, 38]. There was still a need for further investigations regarding the clinical efficacy, safety, and pharmacokinetics of Lapatinib, and thus these were goals for the Phase II/III clinical trials for Lapatinib. The most commonly reported AEs were diarrhea (42%) and rash (31%); diarrhea incidence increased with increasing dose, whereas rash incidence had no correlation with dose regimen [37]. Lapatinib is well tolerated in doses from 500–1600 mg once daily [37].

**Figure 4:**
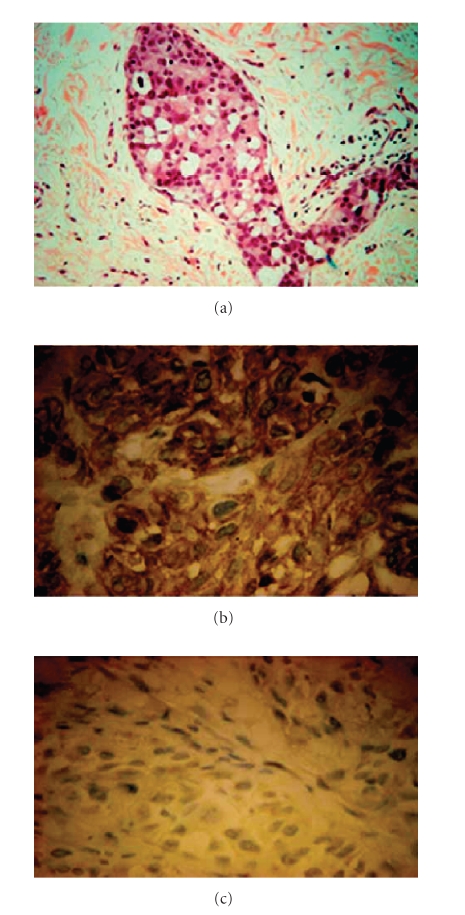
Immunohistochemical staining demonstrating the clinical efficacy of Lapatinib. Figure 4 identifies inhibition of activated, phosphorylated ErbB2/HER-2/neu (p-ErbB2) in a breast cancer patient responding to Lapatinib treatment. (a) Shows a dermal-lymphatic invasion (magenta) that is consistent with recurrent inflammatory breast cancer. (b) and (c) Show further immunohistochemical staining for p-ErbB2 performed on tumor biopsy samples obtained from patient X on days 0 (4B) and 21 (4C) of Lapatinib therapy; note the change in positive staining (brownish-yellow). There is a significant decrease in the activation of p-ErbB2 in response to Lapatinib [37, 38].

Another study from 2006 investigated the dual kinase inhibitor activity of Lapatinib in HER-2-overexpressing breast cancer cells as well as responses of a panel of 31 characterized human breast cancer cell lines to treatment by Trastuzumab, including the use of Trastuzumab-conditioned HER-2(+ve) cell lines [4]. These studies demonstrated four key observations associated with Lapatinib treatment in breast cancer. First, they documented that the anti-proliferative effects of Lapatinib were in fact concentration dependent and were seen in all breast cancer cell lines. Second, they also reported a range of half-maximal inhibitory concentrations for Lapatinib (IC_50_), however, the study demonstrated a significant amount of variation among these values: IC_50_ = 0.010–18.6 *μ*mol/L. Third, these preliminary data were also representative of long-termin vivo Lapatinib treatment regiments for breast cancer; this was ascertained using a 77 consecutive-day Lapatinib treatment schedule in which there was a significant reduction in the volume of human breast cancer xenografts in athymic mice compared with untreated controls [4]. The reduction in tumor volume demonstrated in the aforementioned clinical study with respect to Lapatinib is consistent with the results obtained in the laboratory setting as well (*see Figure* 4) [37, 38]. Lastly, they examined the synergistic effects of a combinatorial therapy of Lapatinib-*plus*-Trastuzumabfor which their results have indeed provided the preliminary data necessary to support the rationale for continuing research regarding the potential of Lapatinib as a combination anti-EGFR therapy with Trastuzumab in HER-2-overexpressing breast cancer and in patients with clinical resistance to Trastuzumab [4]. Thus, this review, in agreement with several earlier reports, supports further investigation of the benefits of Lapatinib as a first-line treatment regimen for early-stage HER-2-overexpressing breast cancer cell lines as well as its use in the treatment of both metastatic and locally advanced breast cancer cases [40].

#### 3.3.2. Erlotinib

Erlotinib treatment is most commonly found in combination with other chemotherapeutic agents, including Capecitabine and Docetaxel. A study researched the additive efficacy of Erlotinib with Capecitabine and Docetaxel [41]. The combined treatment was administered every 3 weeks, with a total of 24 women with MBC; the overall response rate was 67%. The most common treatment-related AEs were skin toxicities and diarrhea. The severe AEs were relatively low, but as the Capecitabine/Docetaxel doses were increased, the rate of grade 3 events also increased. The tolerability of the regimen has been measured and the group reported an established dosage of Erlotinib (100 mg/day), Capecitabine (825 mg/m^2^), and Docetaxel (75 mg/m^2^) in patients with MBC [41].

#### 3.3.3. Gefitinib

Gefitinib has oncebeen approved by FDA as monotherapy for patients with locally advanced or metastatic nonsmall-cell lung cancer (NSCLC) [45]. However, more recently, Gefitinib has been used for the treatment of MBC, including a Phase II study of Gefitinib in combination with Docetaxel as first-line therapy in MBC [42]. In 41 patients, a response rate of 54% (95% CI 45–75%), a stable disease response of 14%, and a progressive disease response of 32% were reported. Grade 3 or 4 toxicities that were observed included neutropenia (49%), diarrhea (10%), acne-like rash (5%), and anemia (2%). Overall, the Gefitinib and Docetaxel combination demonstrated an active and generally well-tolerated regimen in women with MBC who have not been previously treated with metastatic disease [42]. Gefitinib seemed very promising in early clinical phase testing for the treatment of a number of solid tumors, including NSCLC. However, the FDA recently withdrew Gefitinib from its list of clinically effective therapies for NSCLC, but is still currently under critical review in Phase II/III clinical studies for breast cancer: primary, metastatic, and advanced forms.

With respect to ER-HER2/neu cross-talk in ER/HER2/neu(+ve) breast cancer, Gefitinib has demonstrated promising responses [31]. In a tamoxifen-resistant, HER2-overexpressing MCF-7 breast cancer cell line, designated MCF-7/HER2-18, Gefitinib pretreatment was shown to block ER : EGFR receptor cross-talk, reestablish corepressor complexes with tamoxifen-bound ER on target gene promoters, eliminate tamoxifen agonistic effects, and restore tamoxifen antitumor activity both in vitro and in vivo [23].

### 3.4. Combinational Therapies

#### 3.4.1. Lapatinib and Anti-ErbB2 Inhibitors: Trastuzumab

The results of Lapatinib and Trastuzumab monotherapy and combined therapy approaches for treating breast cancer are summarized in Tables 6 and 7. A particular combination therapy study involved the comparison of Lapatinib-*plus*-pAb (where pAb is a rabbit polyclonal antisera generated by vaccination with a human ErbB2 fusion protein) and Lapatinib-*plus*-Trastuzumab [46]. This study showed that Lapatinib-*plus*-Trastuzumab combination therapy had enhanced clinical efficacy compared to both Lapatinib and Trastuzumab monotherapies but had similar efficacy as the secondary combination cocktail of Lapatinib-*plus*-pAb [46]. The Lapatinib-*plus*-Trastuzumab combination therapy also showed both a significant downregulation of survivin, an important prosurvival/antiapoptosis protein, as well as enhanced apoptosis [46]. These conclusions along with the information regarding clinical efficacy and AEs in Table 7 provide sufficient preliminary evidence supporting this synergistic cooperation seen in this combination therapy. It appears that Lapatinib may in fact sensitize the cells to further treatment with Trastuzumab thereby enhancing the individual activity of both drugs.

#### 3.4.2. Lapatinib and Capecitabine

Lapatinib combination therapy with Capecitabine has also shown success in treatment for HER-2(+ve) advanced breast cancer treatment (*see Tables 6 and 7*) [40]. Patients with HER-2(+ve) MBC who had progressed after treatment with regimens that included an anthracycline, a taxene, and Trastuzumab were randomly assigned to receive either Capecitabine alone versus Capecitabine in combination with Lapatinib. The hazard ratio for the independently assessed time to progression was 0.49 (95% CI 0.34 to 0.71; *P* < .001); the median time to progression was 8.4 months in the combination therapy group, whereas 4.4 months in the monotherapy group. The most common AEs were diarrhea, the hand-foot syndrome, nausea, vomiting, fatigue, and rash, varying from grades 1 to 3. In grade 4, diarrhea, fatigue, headache, and dizziness were reported. Discontinuation of treatment due to AEs occurred in 22 women in the combination-therapy group (13%) and in 18 women in the monotherapy group (12%) [40]. However, as previously indicated, these reports are refuted by other sources, which claim no grade 4 AEs and only minor toxicity reports for Lapatinib monotherapy, but there are no other reviews specifically regarding further investigation into the relationship between combinational therapies of Lapatinib-*plus*-Capecitabine, if any does in fact exist, positively or negatively correlated with improved outcome.

Therefore, in concurrence with combinational therapies, a major need clearly exists for further research to be done in this specific area of anti-EGFR therapy. There is a significant deficit in the hypotheses and models that can critically evaluate the data reported thus far on most combinational therapies.

## 4. Conclusion

Breast cancer is a disease that is responsible for approximately 1% of the mortality rate worldwide. The importance of developing new and improved therapies for its treatment is therefore undisputable. Recently, advances in anti-EGFR therapy have given hope to the development of new breast cancer therapies with improved specificity, activity, and safety. Increasingly, there is recognition and acceptance of the unique role anti-EGFR therapy plays in the armamentarium of treatment options available to breast cancer patients. Recently, novel members of this group, such as Lapatinib, have been brought to the forefront of this research as it not only is an extremely effective drug in the clinical setting, but it also serves as an excellent model for the development of future EGFR and/or HER-2 inhibitors. The novel dual kinase inhibitor activity of Lapatinib, which displays tyrosine kinase receptor inhibitory activity against both EGFR and HER-2, is both exciting and intriguing. The unique activity of Lapatinib to inhibit both mechanisms of signaling cascades should be studied extensively in order to improve upon the current model of tyrosine kinase inhibition and its role in anti-EGFR therapy. Other drugs with similar activities to Lapatinib, such as CI-1033, a pan-ErbB tyrosine kinase inhibitor, should also be studied thoroughly in order to identify any important similarities between them and to determine how these crucial factors can perhaps be modified to enhance their activity in future anti-EGFR drug prototypes. Other areas of anti-EGFR therapy that should be investigated include the ability of the various anti-EGFR therapeutic modalities to sensitize cancer cells to other forms of chemotherapy originally considered refractory for an individual patient. This is another extremely important avenue that should be investigated exhaustively.

Although there is much improvement to be done, the wealth of knowledge surrounding these therapies continues to grow (*see Table* 8). This observation, along with recent advances in crystallography and docking techniques, the development of improved high-throughput analyses for identifying novel anti-EGFR activity, as well as advances in DNA/RNA-microarray technology used for classification purposes and extremely useful in the clinical setting, all continue to contribute to the overall understanding and development of these new treatment regiments as well as treating breast cancer as a whole. The design rationale of new anti-EGFR therapies lies in the intimate relationship between the mechanisms of action of current forms of treatment and the structure of the EGFR. We believe that meticulous inspection of the unique intermolecular interactions of these drugs with this receptor and its family members will not only lead to future accomplishments in anti-EGFR therapy but will also increase insight into chemotherapy as a whole for breast cancer.

## Figures and Tables

**Table 1:** Response criteria and evaluation ratings used in the classification of clinical efficacy and safety/toxicity scoring of anti-EGFR therapies for solid tumors. General classification schemes used in review of clinical efficacy and safety, WHO criteria [24].

*General classification schemes used in review of clinical efficacy and safety*:
Objective response and tumor response were evaluated by the WHO Criteria [24].
Adverse events (AEs) were assessed at each cycle using the common toxicity criteria (CTC).
Cardiac failure/cardiac toxicity was graded based on the NYHA classification system.
The Cardiac Review and Evaluation Committee (CREC) evaluates cardiac dysfunction.
*Factors for clinical efficacy of treatment*:
In an intent-to-treat (ITT) population, in order to evaluate the overall response rate of the individual “patient-drug interaction.”
Overall response (OR): complete response (CR) + partial response (PR)
Clinical benefit (CB): complete response (CR) + partial response (PR) + stable disease (SD) ≥ 6 months
Time-to-disease-progression (TTP): the time from randomization (randomized initiation of drug/therapy.
Treatment regimen to be tested) to “disease-progression” or death (whichever event occurs first).

**(a):** 

	No. of patients	
	(*n* = 105)*	
Response	No.	%
CR	2	2
PR	18	17
SD	53	51^†^
CRB (CR + PR + SD > 6 months)	35	33
PD	30	29
ORR	20	19^†^

*Data missing for 2 patients. ^†^One patient with best response of SD in the main study period achieved CR in the 12-month follow-up period. Therefore, in the follow-up analysis, ORR was 20%.

**(b):** 

	No. of patients	
Adverse event	No.	%
Rigors	19	18
Pyrexia	16	15
Headache	11	10
Nausea	10	10
Fatigue	10	10

**Table 3:** Efficacy of Trastuzumab when given in combination with chemotherapy in metastatic breast cancer from Slamon et al. [28].

	Tratuzumab + AC (*n* = 143)	AC alone (*n* = 138)	Trastuzumab + paclitaxel (*n* = 92)	Paclitaxel alone (*n* = 96)	Trastuzumab + chemotherapy (*n* = 235)	Chemotherapy alone (*n* = 234)
Median TTP (months)	7.8	6.1	6.9	3	7.4	4.6
	(*P* = .0004)		(*P* = .0001)		(*P* = .0001)	
Response rate (%)	56	42	41	17	50	32
	(*P* = .0197)		(*P* = .0002)		(*P* < .0001)	
Median duration of response (months)	9.1	6.7	10.5	4.5	9.1	6.1
	(*P* = .0047)		(*P* = .00124)		(*P* = .0002)	
Median TTF (months)	7.2	5.6	5.8	2.9	6.9	4.5
	(*P* = .0014)		(*P* = .0001)		(*P* = .0001)	
1-year survival (%)	83	72	72	60	79	68
	(*P* = .0415)		(*P* = .0975)		(*P* = .008)	
Median survival (months)	26.8	22.8	22.8	18.4	25.4	20.3
					(*P* = .025)	

AC: Anthracycline; TTP: Time to disease progression; TTF: Time-to-treatment failure.

**(a):** 

	Dosing regimen					
	1500 mg once daily		500 mg twice daily		All patients	
	(*n* = 69)		(*n* = 69)		(*N* = 138)	
Patient response	No.	%	No.	%	No.	%
Best response						
* *CR	0	0	0	0	0	0
* *PR	15	22	18	26	33	24
* *Stable disease	40	58	31	45	71	51
* *Progressive disease	8	12	16	23	24	17
* *Unknown	6	9	4	6	10	7
Response rate: CR or PR, %	21.7		26.1		23.9	
* *95% CI	12.7 to 33.3		16.3 to 38.1		17.1 to 31.9	
Odds ratio					0.8	
* *95% CI					0.3 to 1.9	
* * *P*					.691	

**(b):** 

	Dosing regimen					
	1500 mg once daily		500 mg twice daily		All patients	
	(*n* = 69)		(*n* = 69)		(*N* = 138)	
Adverse event*	No.	%	No.	%	No.	%
Diarrhea	24	35	25	36	49	36
* *Grade 1-2	23	33	22	32	45	33
* *Grade 3	1	1	3	4	4	3
Rash	19	29	18	26	37	27
* *Grade 1-2	19	29	17	25	36	26
* *Grade 3	0	0	1	1	1	1
Pruritus	14	20	11	16	25	18
* *Grade 1-2	14	20	11	16	25	18
* *Grade 3	0	0	0	0	0	0
Nausea	9	13	5	7	14	10
* *Grade 1-2	9	13	4	6	13	9
* *Grade 3	0	0	1	1	1	1

*No grade 4 adverse events occurred for these conditions.

**Table 5:** Clinical efficacy of Trastuzumab and Lapatinib as monotherapy agents for metastatic breast cancer [26, 27, 30, 39].

Study	No. of patients	Initial and following dose	OR (%)	Median TOP and range (months)
Trastuzumab				
Baselga et al. [27]	105	8 mg/kg, 6 mg/kg triweekly	19	3.4 (range 0.6–23.6)
Cobleigh et al. [26]	222	4 mg/kg, 2 mg/kg weekly	15	3.1 (range 0–≥28)
Vogel et al. [39]	114	4 mg/kg, 2 mg/kg weekly	26	3.8 (range 3.3–5.3)
		Or 8 mg/kg, 4 mg/kg weekly		
Lapatinib				
Gomez et al. [30]	69	1500 mg once daily	24	4.4 (range 0.5–23)
		Or 500 mg twice daily		

OR: Overall response rate; TOP: Time to progression. To date, most lapatinib therapies are still in progress and currently being evaluated.

**Table 6:** Overall response for Trastuzumab, Lapatinib, Erlotinib, and Gefitinib combination therapies with chemotherapeutic agents [34, 40–43].

Study	No. of patients	Chemotherapy	Dose	OR (%)
Trastuzumab				
Slamon et al. [34]	143	Doxorubicin	Trastuzumab (4 mg/kg initial dose, 2 mg/kg weekly)	56
			Doxorubicin (60 mg/m^2^)	
	92	Paclitaxel	Trastuzumab (4 mg/kg initial dose, 2 mg/kg weekly)	41
			Paclitaxel (175 mg/m^2^)	
Marty et al. [43]	186	Docetaxel	Trastuzumab (4 mg/kg initial dose, 2 mg/kg weekly)	34
			Docetaxel (100 mg/m^2^ triweekly)	
Lapatinib				
Geyer et al. [40]	163	Capecitabine	Lapatinib (1250 mg/day)	22*
			Capecitabine (2000 mg/m^2^)	
Erlotinib				
Twelves et al. [41]	24	Capecitabine, docetaxel	Erlotinib (100 mg/day)	68
			Capecitabine (825 mg/m^2^)	
			Docetaxel (75 mg/m^2^)	
Gefitinib				
Ciardiello et al. [42]	41	Docetaxel	Geftinib (250 mg/day)	54
			Docetaxel (75 mg/m^2^ or 100 mg/m^2^)	

OR: Overall response rate. *Study was performed in women with HER2-positive metastatic breast cancer that has progressed after trastuzumab-based therapy.

**Table 7:** Efficacy end points in intent-to-treat population, adapted from Geyer et al. [40].

End point	Lapatinib plus capecitabine	Capecitabine alone	Hazard ratio	*P*-value
	(*N* = 163)	(*N* = 161)	(95% CI)	
Median time to progression—mo	8.4	4.4	0.49 (0.34–0.71)	<.001^†^
Median progression-free survival—mo	8.4	4.1	0.47 (0.33–0.67)	<.001^†^
Overall response—% (95% CI)	22 (16–29)	14 (9–21)		.09^‡^
* *Complete response—no. (%)	1 (<1)	0 (0)		
* *Partial response—no. (%)	35 (21)	23 (14)		
Clinical benefit—no. (%)	44 (27)	29 (18)		
Death—no. (%)	36 (22)	35 (22)		

End Points are based on evaluation by the independent review committee under blinded conditions. ^†^The *P*-value was calculated with the log-rank test. ^‡ ^The *P*-value was calculated with Fisher's exact test.

**Table 8:** Summary of Anti-EGFR therapy agents. The *5* anti-EGFR therapy drugs discussed in this review: these 5 drugs are currently being used or in clinical phase testing for anti-EGFR therapy of breast cancer. All of these agents are either already being used in the clinical setting or are in Phase III clinical development.

Drug name	Other names for the drug	Classification of drug	Target receptor(s) of drug	Special cancer types and efficacy	Important comments	Drug manufacturer
*Cetuximab*	*Erbitux* (humanized form of the murine MoAb: C225)	MoAb (chimeric IgG_1_)	Blocks EGFR;	A large variety of solid tumors: -CRC/mCRC; -SCCHN;	Most widely used anti-EGFR monoclonal antibody used in solid tumor therapy (07/2007) [44];	ImClone Systems, Inc., Princeton, NJ. & NY, NY.
*Trastuzumab*	*Herceptin*	MoAb (human IgG_1_)	Blocks HER2/neu;	Mostly widely used MoAb in treating HER2+ -overexpressing cases of BC;	Extremely important drug in breast cancer;	F. Hoffmann-La Roche Ltd, Basel, Switzerland.
*Erlotinib*	*Tarceva* (*OSI-774*)	smTKI	Inhibition of EGFR;	Solid tumor therapy: -Pancreatic cancer; -NSCLC (recent);	Nothing unique;	Genetech, Inc., South San Fransisco, CA.
*Gefitinib*	*Iressa* (*ZD1839*)	smTKI -anilinoquinazoline	Inhibition of EGFR;	Previously used for NSCLC-currently w/d by FDA;	Recently withdrawn by FDA for treatment of NSCLC;	AstraZeneca Pharmaceuticals, Wilmington, DE.
				In clinical phase testing for BC as well as other metastatic & advanced cancers;		
*Lapatinib*	*Tykerb* (*GW572016*)	smTKI	Both EGFR and HER2/neu; (Dual-TKI Action)	Solid tumor therapy: -BC.	Extremely important smTKI in current BC treatment.	Glaxo-Smith-Kline, Philadelphia, PA.

MoAb: Monoclonal antibody; EGFR: Epidermal growth factor receptor (ErbB1); HER-2/neu: Human epidermal growth factor receptor 2; smTKI: Small molecule tyrosine kinase inhibitor; w/d: Withdrawn; NSCLC: Nonsmall cell lung cancer; BC: Breast cancer; CRC: Colorectal cancer; mCRC: Metastatic colorectal cancer; SCCHN: Squamous cell carcinoma of the head and neck.
